# The Functional Roles and Regulation of Circular RNAs during Cellular Stresses

**DOI:** 10.3390/ncrna8030038

**Published:** 2022-05-27

**Authors:** Yueh-Chun Lee, Wei-Yu Wang, Hui-Hsuan Lin, Yi-Ren Huang, Ya-Chi Lin, Kuei-Yang Hsiao

**Affiliations:** 1 Department of Radiation Oncology, Chung Shan Medical University Hospital, Taichung 40201, Taiwan; cshy1014@csh.org.tw; 2 School of Medicine, Chung Shan Medical University, Taichung 40201, Taiwan; 3 Division of Hemato-Oncology, Department of Internal Medicine, Ditmanson Medical Foundation Chia-Yi Christian Hospital, Chia-Yi City 60002, Taiwan; 07523@cych.org.tw; 4 Ph.D. Program in Tissue Engineering and Regenerative Medicine, National Chung Hsing University, Taichung 40227, Taiwan; d109001705@mail.nchu.edu.tw; 5 Institute of Biochemistry, College of Life Sciences, National Chung Hsing University, Taichung 40227, Taiwan; g110058003@mail.nchu.edu.tw; 6 Department of Plant Pathology, College of Agriculture and Natural Resources, National Chung Hsing University, Taichung 40227, Taiwan; kd04837@dragon.nchu.edu.tw; 7 Department of Medical Laboratory Science and Biotechnology, Asia University, Taichung 41354, Taiwan; 8 Ph.D. Program in Translational Medicine, College of Life Sciences, National Chung Hsing University, Taichung 40227, Taiwan; 9 Rong Hsing Research Center for Translational Medicine, College of Life Sciences, National Chung Hsing University, Taichung 40227, Taiwan; 10 Bachelor Program of Biotechnology, College of Agriculture and Natural Resources, National Chung Hsing University, Taichung 40227, Taiwan

**Keywords:** circRNA, backsplicing, DNA damage response, genotoxic stress, chemoresistance, hypoxia, heat shock, m6A

## Abstract

Circular RNAs (circRNAs) are a novel class of regulatory RNA involved in many biological, physiological and pathological processes by functioning as a molecular sponge, transcriptional/epigenetic/splicing regulator, modulator of protein–protein interactions, and a template for encoding proteins. Cells are constantly dealing with stimuli from the microenvironment, and proper responses rely on both the precise control of gene expression networks and protein–protein interactions at the molecular level. The critical roles of circRNAs in the regulation of these processes have been heavily studied in the past decades. However, how the microenvironmental stimulation controls the circRNA biogenesis, cellular shuttling, translation efficiency and degradation globally and/or individually remains largely uncharacterized. In this review, how the impact of major microenvironmental stresses on the known transcription factors, splicing modulators and epitranscriptomic regulators, and thereby how they may contribute to the regulation of circRNAs, is discussed. These lines of evidence will provide new insight into how the biogenesis and functions of circRNA can be precisely controlled and targeted for treating human diseases.

## 1. Introduction

Circular RNA is a class of regulatory RNA with a circular configuration, and is produced by backsplicing, in which the downstream splice donor reacts with the upstream splice acceptor by canonical spliceosome machinery. Since its first discovery in mammalian cells in 1979 [1] and the rediscovery of its molecular function and wide distribution in various tissues in 2012 [2,3,4,5], great attention has been paid to the molecular functions of circRNA, such as the microRNA (miRNA)/RNA-binding protein (RBP) sponge [4,6,7,8], transcriptional/epigenetic regulator [9,10], splicing regulator [11,12], modulator for protein–protein interactions [13,14,15] and templates for encoding proteins [16,17,18].

**Figure 1:**
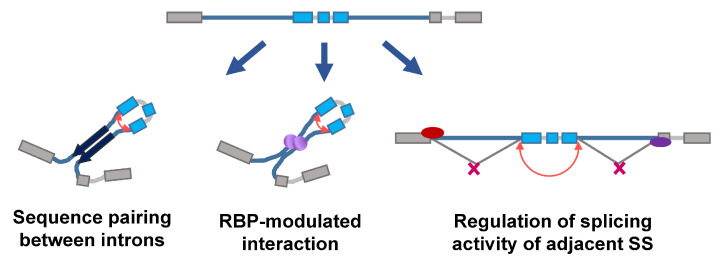
Different models controlling backsplicing. The given pre-spliced RNA (**top**) consists of introns (thin lines; blue ones for flanking long introns), circularizable exons (blue boxes) and the exons for linear splicing (grey boxes), and may undergo the following processes for backsplicing. The left panel shows the sequence-pairing dependent mechanism. A pair of arrows indicates the complementary sequences in the flanking long introns for circularizable exons. The red curved arrow indicates where the backsplicing takes place. Similarly, the interaction between flanking introns can be mediated by RBPs (**middle** panel, purple spheres). Alternatively, the splicing modulators (**right** panel, red or purple ovals) occupy the splicing signals (SS) and decrease the availability of splice donors in the upstream intron and/or the availability of splice acceptors in the downstream intron, inhibiting linear splicing and thereby favoring backsplicing.

It is known that circRNAs are transcribed from both the exonic and intronic regions of coding or noncoding genes [19,20,21,22]. Several genome-wide analyses reported that circRNAs originate from virtually any genomic locus, such as intergenic, intronic, coding region, 5′- and 3′-untranslational regions (UTRs) [23,24]. Despite the diverse loci/regions of origin, the majority of circRNAs are produced by the unique process termed ‘backsplicing’, in which a downstream splice donor joins an upstream splice acceptor with no preference for U2 or U12 spliceosome [5,25]. However, there are a few molecular properties/events favoring the occurrence of backsplicing. First, the exons flanked by large introns tend to be circularized [2,5,26,27] (Figure 1, top). Second, the repetitive sequences or any complementary sequences in the flanking introns would bring the downstream splice donor and upstream splice acceptor to a proximity, allowing the backsplicing to take place [5,9,28,29] (Figure 1, left). In an analogy, RBPs antagonizing complementary pairing hinder the efficiency of backsplicing, while RBPs favoring the interaction between flanking introns enhance backsplicing [30] (Figure 1, middle). Lastly, the lost/masking of the splice donor of the upstream flanking intron or splice acceptor of the downstream flanking intron also increases the frequency of backsplicing (Figure 1, right). The cells should respond to the external stimulation properly, and deregulated responses to cellular stresses typically cause the development of human diseases. In this mini-review, we would like to focus on the potential mechanisms underlying how cells modulate the process of backsplicing in response to or during the cellular stresses. The presentation of these lines of clues would help to bridge the external stimuli, backsplicing, and finally the biogenesis and functions of circRNAs, shedding light on the identification of potential targets for treating human diseases.

## 2. Roles of CircRNAs in Cellular Stress

### 2.1. Roles and Regulation of CircRNAs in Response to Genotoxic Stress

**Figure 2:**
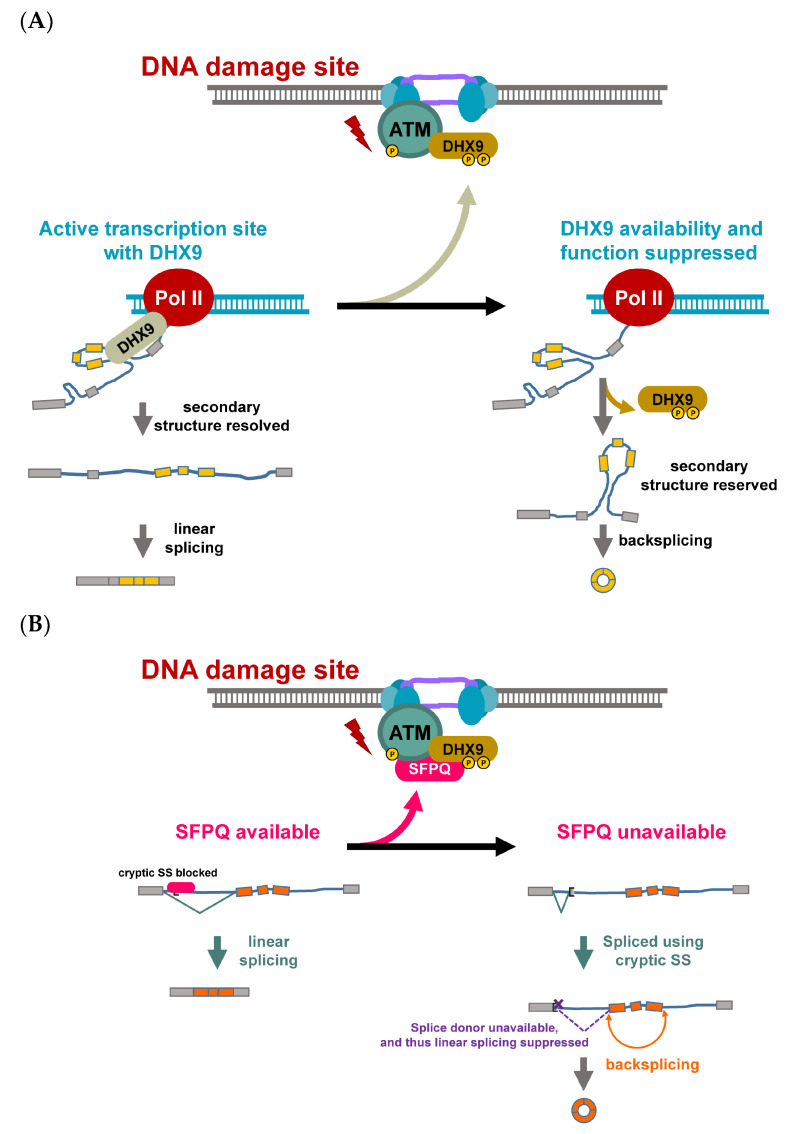
DNA damage response controls the function and availability of RBP for regulation of backsplicing. (**A**) In the absence of DNA damage, DHX9 works with transcription machinery for resolving the secondary structure of nascent RNA, and thereby keeps a low frequency of backsplicing (**bottom left**). Upon double-stranded DNA breaks (**top**), the PI3KKs (ATM for example) are recruited to the damage sites, accumulated and autophosphorylated for its maximal kinase activity and for recruiting other DDR proteins. DHX9 is recruited to the damage site and becomes phosphorylated near to its double-stranded RNA binding domain, hindering its binding to the double-stranded RNA substrate. The unresolved pairing between upstream and downstream introns allows the backsplicing to take place (**bottom right**). (**B**) In a similar fashion, SFPQ forms a complex with DHX9 upon genotoxic stress. When SFPQ sits on the cryptic splice site in the upstream intron of circularizable exons (orange boxes), SFPQ suppresses the linear splicing, which uses the cryptic site (**bottom left**). However, when SFPQ becomes unavailable due to genotoxic stress-induced relocation to DNA damage sites, the splicing uses up the splice signal, making backsplicing the only option for the rest of the molecule (**bottom right**).

DNA damage poses a significant threat to the cells, and thus it is critical for cells to properly respond to the DNA damage. One of the early molecular events of DNA damage is mediated by phosphatidylinositol 3-kinase-related kinase (PI3KK) activation. In addition to DNA repair, the activation of PI3KK coordinates global transcriptional repression [31,32,33,34]. DExH-Box helicase 9 (DHX9), also known as RNA helicase A, is associated with active transcription machinery and is involved in resolving the secondary structure during active transcription [35,36] (Figure 2A, left), while its helicase activity negatively regulates the formation of circRNA [36]. Interestingly, DHX9 also plays important roles during DNA damage response and is associated with factors related to DNA damage [37,38] (Figure 2A, top). It was shown that the depletion of DHX9 using small interfering RNA decreases the levels of a few circRNAs [36], and thus, factors inhibiting DHX9 activity may potentially facilitate circRNA backsplicing. Intriguingly, DHX9 was post-translationally regulated by DNA damage-induced ATM/ATR activation [39,40] (Figure 2A, top and right), and the phosphorylation sites located adjacent to the RNA binding motif. This suppressive phosphorylation promotes the level of an oncogenic circRNA, CCDC66, which governs a subset of oncogenes contributing to the development of chemoresistance [7,40]. The aberrant induction of circRNAs is a feature of chemo-/radio-resistant colorectal cancer (CRC). It has been reported that a group of circRNAs was upregulated in fluorouracil/radio-resistant CRC consistent with the observation in oxaliplatin-resistant CRC [40,41]. In both platinum-based chemo-resistant gastrointestinal tract-derived tumors, the patients with higher levels of these circRNAs had a poorer prognosis, and the induction of the chemoresistance-regulated circRNAs contributed to the metastatic features of the tumors [7,40,42]. In agreement with the role of DHX9 in DNA damage response, DHX9 interacting partners, splicing factor proline/glutamine rich (SFPQ) and non-POU domain-containing octamer binding protein (NONO), were also recruited to the DNA damage site in an ATM-dependent manner [43] (Figure 2B). Functionally, SFPQ hinders the activity of the cryptic splicing signal, regulating the availability of the splicing signal for backsplicing [44]. The change of the subnuclear localization of SFPQ and NONO may alter their availability for regulating splicing. It has been reported that the absence/unavailability of SFPQ in the upstream intron of circularizable exons promoted the backsplicing efficiency of a particular circRNA with a long intron, but no proximal inverted Alu elements [44], implying that SFPQ may be involved in the regulation of genotoxic stress-induced circRNA biogenesis.

The exact roles of circRNA have not been thoroughly explored. However, some pioneer studies of noncoding RNA may point out the direction. For example, a group of small non-coding RNAs, termed DNA damage-response RNA (DDRNA), were produced by DICER and DROSHA in response to DNA damage, and contributed to the accumulation of MDC1 and 53BP1 in the late stage of the DNA damage response [45]. lncRNA in nonhomologous end joining (NHEJ) pathway 1 (LINP1) served as a platform for Ku80 and DNA-dependent protein kinase catalytic subunits (DNA-PKcs), promoting the activity of NHEJ [46]. In contrast, small Cajal body-specific RNA 2 (scaRNA2) constrained the activity of DNA-PK through binding to the catalytic subunit, and thus, scaRNA2 weakens its interaction with the Ku70/80 subunits, as well as with the LINP1 lncRNA [47]. Whether circRNA is involved in this fine regulation warrants further investigation.

### 2.2. Modulation of CircRNA under Hypoxic Stress

#### 2.2.1. Hypoxia-Regulated CircRNAs

Hypoxia, a condition in which cells are deprived of an adequate oxygen supply, is one of the most challenging stresses to tissues, and plays vital roles in both physiological and pathological processes such as tumorigenesis and diseases in various tissues/organs [48,49,50,51,52]. The post-translational and transcriptional regulation of hypoxia-inducible factor (HIF) have been extensively investigated [53], but the roles of circRNA in response to hypoxia is yet to be explored. Hypoxia-regulated circRNAs were identified through transcriptomic analyses in a handful pioneer studies [54,55]. Several hypoxia-induced circR-

**Figure 3:**
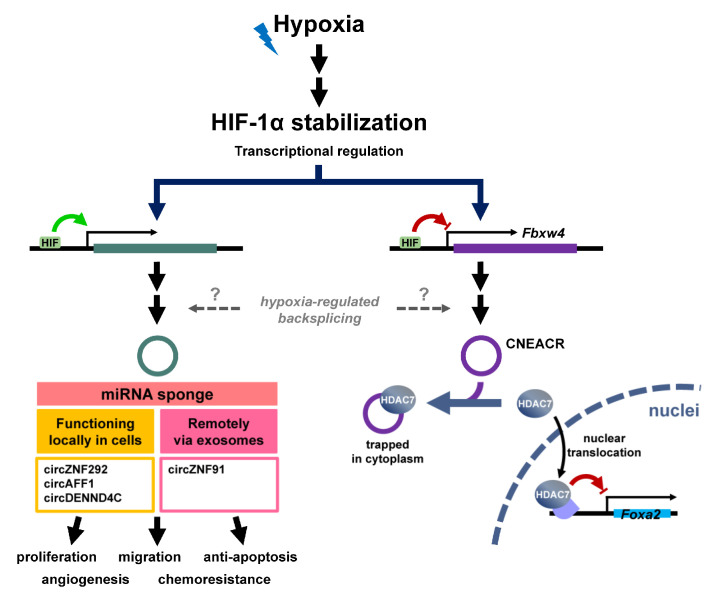
HIF-mediated transcriptional regulation of circRNAs. The majority of circRNA expression in response to hypoxia is mediated through HIF-1α, which is stabilized by hypoxic stress (**top**). Through binding to the promoter of target genes, HIF-1α either activates or suppresses the transcription. A group of circRNA is upregulated via transcriptional activation, and mainly functions as a miRNA sponge. CircZNF91 is incorporated into exosomes and delivered to remote sites where it exerts its activity as an miRNA sponge. Nevertheless, CNEACR binds and sequesters HDAC7 in cytoplasm in normoxia, while HIF-1α-inhibited CNEACR expression allows HDAC7 to be released and to translocate to nuclei in hypoxia. HDAC7 represses the expression of Foxa2, relieving the expression of Foxa2-inhibited target genes.

NAs such as cireZNF292, circAFF1 and circDENND4C have been identified (Figure 3, left). The upregulation of these genes is prone to result from the transcriptional activation, but not the alteration of backsplicing efficiency. Among these circRNAs, circZNF292 was shown to promote endothelial cell proliferation and sprouting through a non-miRNA sponging activity. An independent study using models of breast cancer also reported that the level of circDENND4C was upregulated by hypoxic stress, and was positively correlated to the HIF-1α mRNA level and tumor size [28], implying the importance of transcriptional activation by HIF-1α under hypoxia for circDENND4C induction. In spite of the uncharacterized molecular functions of these hypoxia-regulated circRNAs, a cardiac- necroptosis-associated circRNA (CNEACR) originating from exon 2 to 5 of *Fbxw4* was identified from a mouse model of ischemia/reperfusion [56] (Figure 3, right). The level of CNEACR was downregulated by ischemia/reperfusion in mice, and the same pattern was confirmed in the cell culture system. The expression of CNEACR was specific to myocytes compared to fibroblasts, and resided more in cytoplasm than nuclei. The overexpression of CNEAR attenuated hypoxia/reoxygenation-induced cell death. It was observed that CNEAR interacts with histone deacetylase 7 (HDAC7) by using biotin pulldown assay and trapping HDAC7 in cytoplasm. Mechanically, the trapping of HDAC7, a transcriptional co-repressor, in cytoplasm relieved the suppression of Foxa2 and facilitated the induction of Foxa2-regulated receptor-interacting protein kinase 3 (RIPK3). This particularly widened the horizon regarding how circRNA may function in addition to miRNA sponges in physiological and pathological conditions. Nevertheless, it has been reported that circRNA may be used for inter-cellular communication by incorporation into exosomes [57,58]. Among these exosomal circRNAs, the level of circZNF91 was elevated by HIF-1α-mediated transcriptional activation, and was shown to antagonize the activity of miR-23b-3p, protecting SIRT1 mRNA from degradation and contributing the development of gemcitabine resistance [59].

#### 2.2.2. Players Regulate Function and Abundance of CircRNAs in Hypoxia

Although the mechanisms underlying hypoxia-regulated circRNA biogenesis and functions have not yet been fully explored, there are a few studies that have attempted to demonstrate potential mechanisms. A comprehensive transcriptomic study indicated that the binding motifs of heterogeneous nuclear ribonucleoprotein C (hnRNPC), human antigen R (HuR) and poly(A)-binding protein 4 are enriched in the flanking introns of hypoxia-regulated circRNAs [55]. Although none of these RBPs are readily linked to circRNA biogenesis, there are some clues linking the regulation of backsplicing and hypoxia. For example, the interaction of hnRNPC with DHX9 and competition with splicing factor U2AF 65 kDa subunit (U2AF65) in Alu element make hnRNPC a potential regulator for circRNA biogenesis under hypoxia [36,60]. The role of hnRNPC in controlling intron pairing and backsplicing is supported by a study showing that the knockdown of hnRNPC increased the abundance of double-stranded RNA regions [61]. This suppressive role of hnRNPC on double-stranded RNA formation may be partially contributed to by the ability of hnRNPC to interact with DHX9 [36]. In addition to resolving the RNA pairing, completion between hnRNPC and U2AF65 for U tract within Alu elements suppresses the splicing activity [60], and thus potentially inhibits backsplicing in the context of circRNA biogenesis. In addition, it has been reported that the level of HuR is modulated by hypoxic stress [62], and recognizes RNA motifs via the formation of homodimer [63], implying that the backsplicing efficiency may be potentially modulated by the hypoxia-regulated availability of HuR.

**Figure 4:**
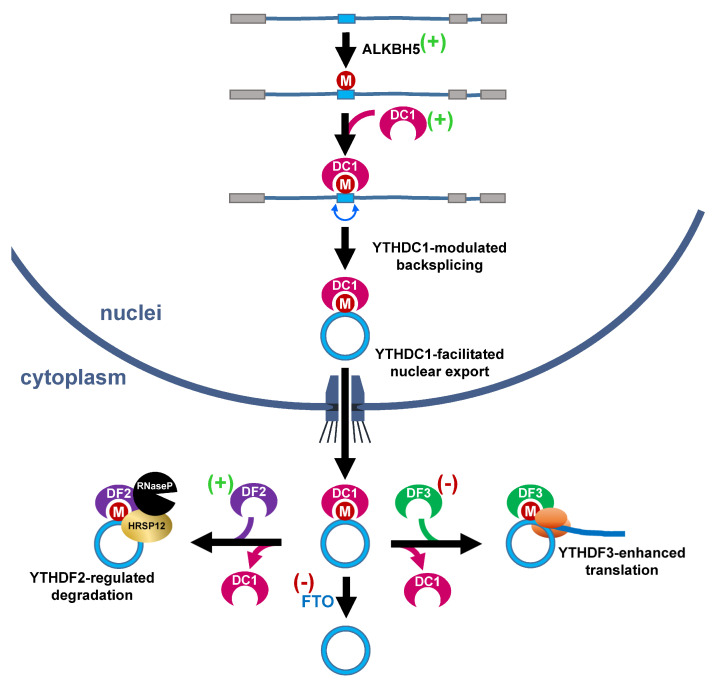
Potential roles of hypoxia-responsive m6A effectors in regulation of circRNA functions. The deposition of m6A on exons by m6A writers (such as ALKBH5) allows the binding of YTHDC1 (DC1), which promotes the backsplicing of circZNF609. In addition, YTHDC1 also helps the nuclear export of circRNA. In cytoplasm, the m6A-harboring circRNA is recognized by YTHDF3 (DF3) and proceeded to translation, or alternatively bound by YTHDF2 (DF2) and targeted for degradation. The m6A eraser, FTO, eventually removes the modification. (+): reported to be upregulated by hypoxic stress; (−): suppressed by hypoxia.

N^6^-methyladenosine (m6A), one of the most prevalent, abundant and conserved modifications identified in eukaryotic RNAs, was recently identified on circRNA [64,65] and serves as another link to hypoxia-regulated circRNA functions and homeostasis. The abundance of m6A is dynamically regulated by a dozen m6A methyltransferases, such as methyltransferase-like 3/14/16 [66,67,68], zinc finger CCCH-type containing 3 [69], RNA binding motif protein 15 [70], WT1-associated protein [71,72] and vir-like m6A methyltransferase-associated [72], and demethylases, fat mass and obesity-associated (FTO) and AlkB homolog 5 (ALKBH5) [73,74]. Different effectors/readers include YTH domain-containing family 1/2/3 (YTHDF1/2/3), YTH domain-containing 1/2 (YTHDC1/2), insulin-like growth factor 2 mRNA-binding protein 1/2/3 (IGF2BP1/2/3) and hnRNPA2B1 grant m6A multiple cellular/molecular functions. The deposition of m6A on circRNA differentially controls various molecular events such as nuclear export, degradation, biogenesis and translation by a distinct m6A reader [64,75,76,77,78]. For example, the deposition of m6A in exon followed by the recognition of YTHDC1 promotes the backsplicing of circZNF609 and nuclear export of circNSUN2 in nuclei, while recognition by YTHDF3 in cytoplasm increases translation [75,77] (Figure 4, top). Through recognition by a distinct m6A reader, YTHDF2, which works with heat-responsive protein 12 and RNase P, a subset of m6A-containg circRNAs, was destabilized [76]. Intriguingly, the expression of many m6A regulators was regulated by hypoxic stress. The expression of m6A demethylase, FTO, was suppressed by hypoxia [79], while the levels of ALKBH5, YTHDC1, YTHDF1 and YTHDF2 were upregulated [80,81,82,83,84] (Figure 4, indicated by (+) or (−)). Most of these cases were direct targets of the hypoxia-inducible factor, while YTHDF1 was post-transcription-ally regulated by hypoxia-induced miR-16-5p. These lines of evidence suggest that the hypoxia-regulated m6A dynamics may control functions of a subset of circRNAs. Along with the hypoxia-m6A-circRNA regulatory axis, circRNA also controls the level of HIF-1α. For example, it was reported that circERBIN promotes the expression of HIF-1α through upregulation of the cap-independent translation of HIF-1α by suppressing miRNAs targeting 4EBP-1 [85]. Taken together, this evidence highlights the critical roles of circRNA during hypoxic stress.

## 3. Heat Shock Stress

**Figure 5:**
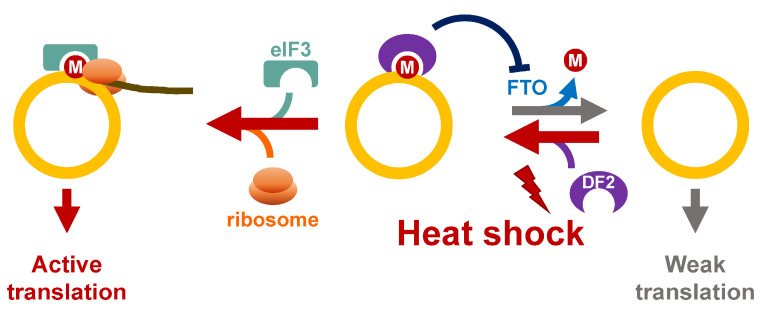
Potential roles of m6A in regulation of circRNA translation in response to heat shock. Upon heat stress, YTHDF2 (DF2) binds m6A-modified circRNA, and prevents FTO-mediated demethylation. The ribosomes are recruited to circRNA through eIF3/YTHDF2.

The heat shock response is well studied in aspects of both transcriptional and translational regulation. However, whether the functions or expression of circRNA are controlled under such circumstances remains largely unknown. Recently, the coding potential of circRNA has begun to receive more attention. One of the cap-independent translations of circRNA is mediated by m6A [77] (Figure 5). It has been reported that m6A reader—YTHDF2—translocates to the nuclei and protects the m6A within 5′-UTR of mRNA from FTO-mediated demethylation and promotes translation through the recruitment of eukaryotic initiation factor 3 (eIF3) in response to heat shock [86,87]. Consistent with the role of the m6A-stimulating cap-independent translation of mRNA, the m6A-mediated translation of circRNA is enhanced by heat shock [17,64], implying that YTHDF2 and/or eIF3 may be potential players for regulating the coding activity of circRNA under heat shock stress.

## 4. Closing Remarks

The regulation of circRNA under genotoxic stress, hypoxia and heat shock stress is discussed in the current article. Although many progressions have been made in the last decade since the re-discovery of circRNA regarding its functions and distribution in 2012 [2,3,4,5], the diverse mechanisms underlying the regulation of backsplicing still remain largely uncharacterized. In this mini-review, how the known effectors under three of the major cellular stresses potentially link to backsplicing modulators were outlined and discussed. These materials should prompt researchers to identify the current gaps in circRNA regulation, and hopefully stimulate the investigation for paving new roads for the development of novel strategies to treat human diseases.
